# Supplementary figures and images for: Aging, Rather than Genotype, Is the Principal Contributor to Differential Gene Expression Within Targeted Replacement APOE2, APOE3, and APOE4 Mouse Brain
Source: Brain Sci. 2025 Oct 17;15(10):1117. doi: 10.3390/brainsci15101117 (PMC12563672; doi:10.3390/brainsci15101117)

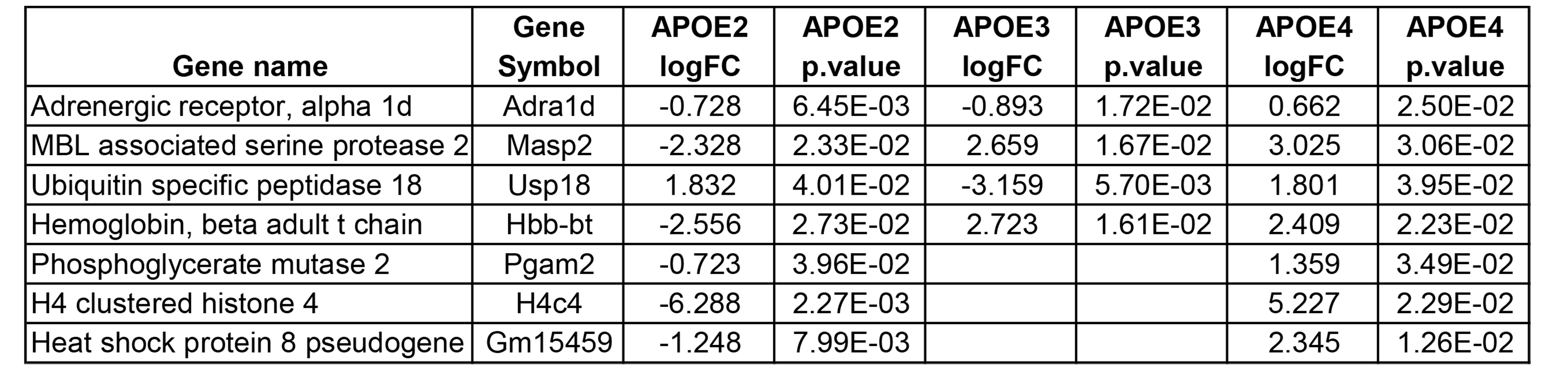

Supplement: Supplementary file 1 [file brainsci-15-01117-s001.zip › Labuza_ApoE_Suppl Table S1.tif]

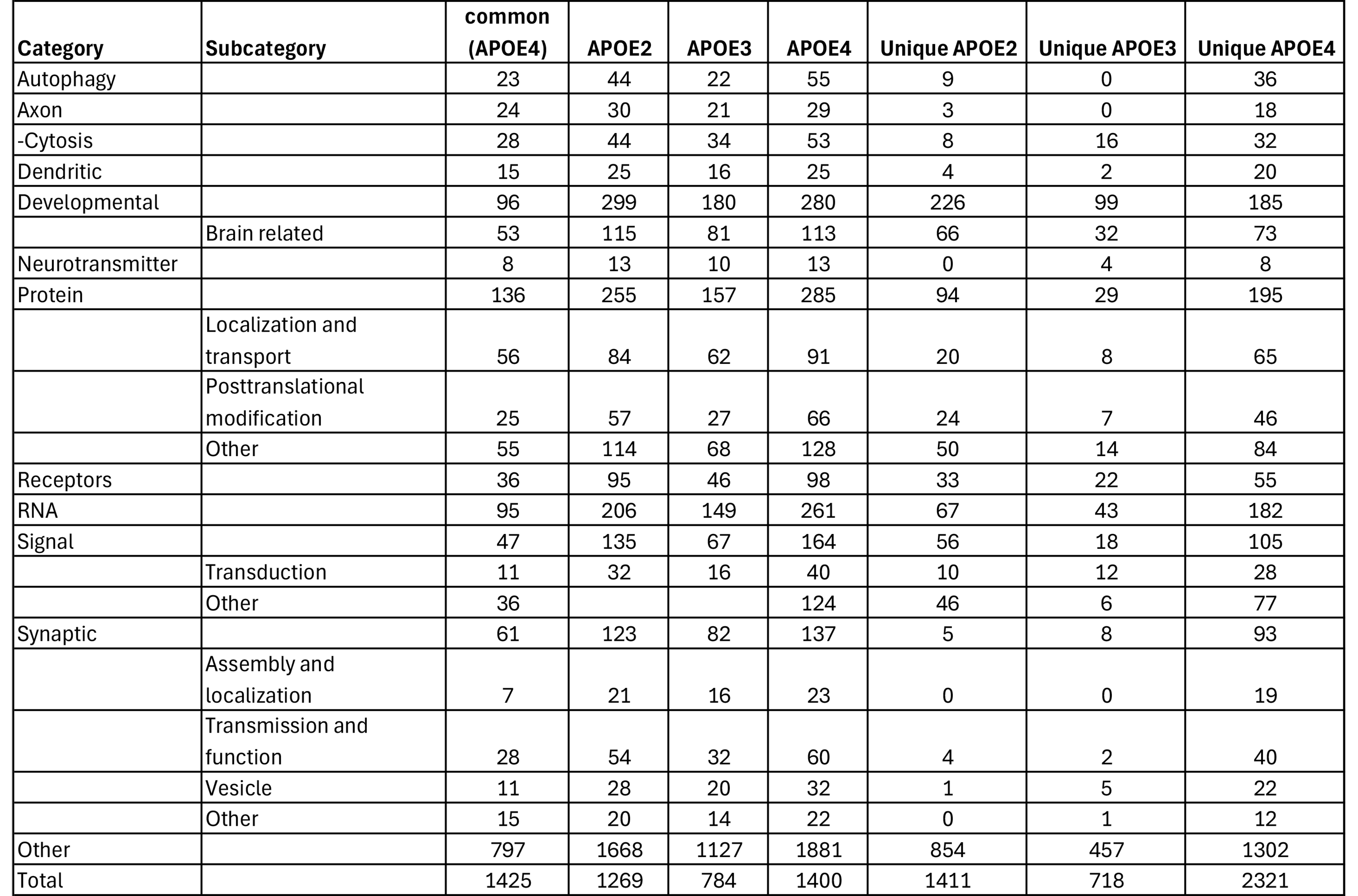

Supplement: Supplementary file 1 [file brainsci-15-01117-s001.zip › Labuza_ApoE_Suppl Table S2.tif]

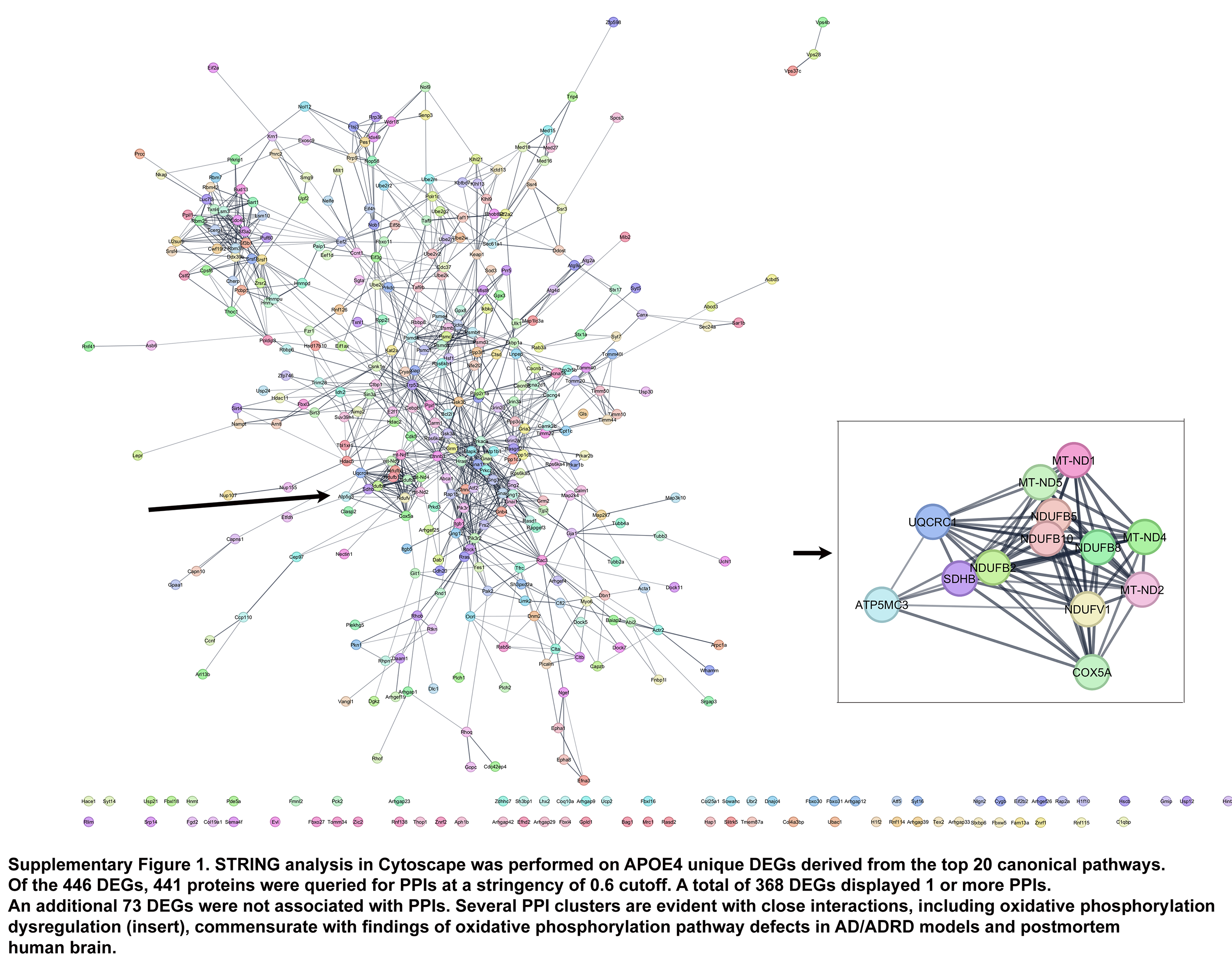

Supplement: Supplementary file 1 [file brainsci-15-01117-s001.zip › Supplementary figure S1_update.tif]
